# CT-based radiomics improves survival prediction in colorectal liver metastases: beyond clinical scores

**DOI:** 10.1007/s13304-026-02649-z

**Published:** 2026-06-17

**Authors:** Angela Ammirabile, Gilda Matteucci, Francesco Fiz, Elisa Ragaini, Sofia Moroni, Ezio Lanza, Lara Cavinato, Jacopo Galvanin, Chiara Masci, Guido Costa, Andrea Laghi, Guido Torzilli, Francesca Ieva, Luca Viganò

**Affiliations:** 1 Department of Biomedical Sciences, Humanitas University, Via Rita Levi Montalcini 4, Pieve Emanuele, Milan, 20072 Italy; 2 Department of Diagnostic and Interventional Radiology, IRCCS Humanitas Research Hospital, Milan, Italy; 3 MOX Laboratory, Department of Mathematics, Politecnico di Milano, Milan, Italy; 4 Nuclear Medicine Unit, Department of Diagnostic Imaging, Ente Ospedaliero Ospedali Galliera, Genoa, Italy; 5 Division of Hepatobiliary and General Surgery, Department of Surgery, IRCCS Humanitas Research Hospital, Milan, Italy; 6 Hepatobiliary Unit, Department of Minimally Invasive General and Oncologic Surgery, Humanitas Gavazzeni University Hospital, Viale M. Gavazzeni 21, Bergamo, 24125 Italy

**Keywords:** Radiomics, Colorectal liver metastases, Overall survival, Colorectal cancer, Computed tomography

## Abstract

Surgery with perioperative chemotherapy offers a potentially curative treatment for colorectal liver metastases (CRLM). Selection of candidates for resection relies on survival prediction, but available prognostic factors have limited reliability. This study evaluated the potential of preoperative CT-based radiomics to predict overall survival, focusing on the impact of the CT–surgery interval and peritumoral tissue analysis. All consecutive patients undergoing resection for CRLM (2010–2020) with contrast-enhanced CT performed ≤ 60 days before surgery and at least one CRLM ≥ 10 mm were considered. Manual tumor segmentation (Tumor-VOI) and automatic 5-mm peritumoral expansion (Margin-VOI) were performed on portal phase images. From each VOI, 110 IBSI-compliant radiomic features were extracted. Three prediction models were developed: Clinical, Clinical+Tumor-radiomics, Clinical+Tumor/Margin-radiomics. Features selection was performed using Boruta algorithm, followed by Random Forest classification with 10-fold cross-validation. Model performance was evaluated in the entire cohort and in patients with CT-surgery interval ≤ 30 days. 306 patients were included (mean age 63 years; 187 men). Five-year survival was 40.9% (mean follow-up 34 months). At internal validation, the clinical model achieved C-index = 0.629. Radiomics provided modest improvement in the entire cohort, with greater impact in the 212 patients with a CT-surgery interval ≤ 30 days: the Clinical+Tumor-radiomics model reached C-index = 0.691, increasing to 0.717 with Margin-VOI features. Clinical–radiomic models outperformed established scores (Fong, GAME, RAS-mutation clinical scores; C-indices range = 0.502–0.593). Radiomic features of CRLM and peritumoral tissue extracted from preoperative CT improve survival prediction beyond conventional clinical scores. A CT-surgery interval of ≤ 30 days appears essential to optimize model performance.

**Graphical abstract:**

**Figure:**
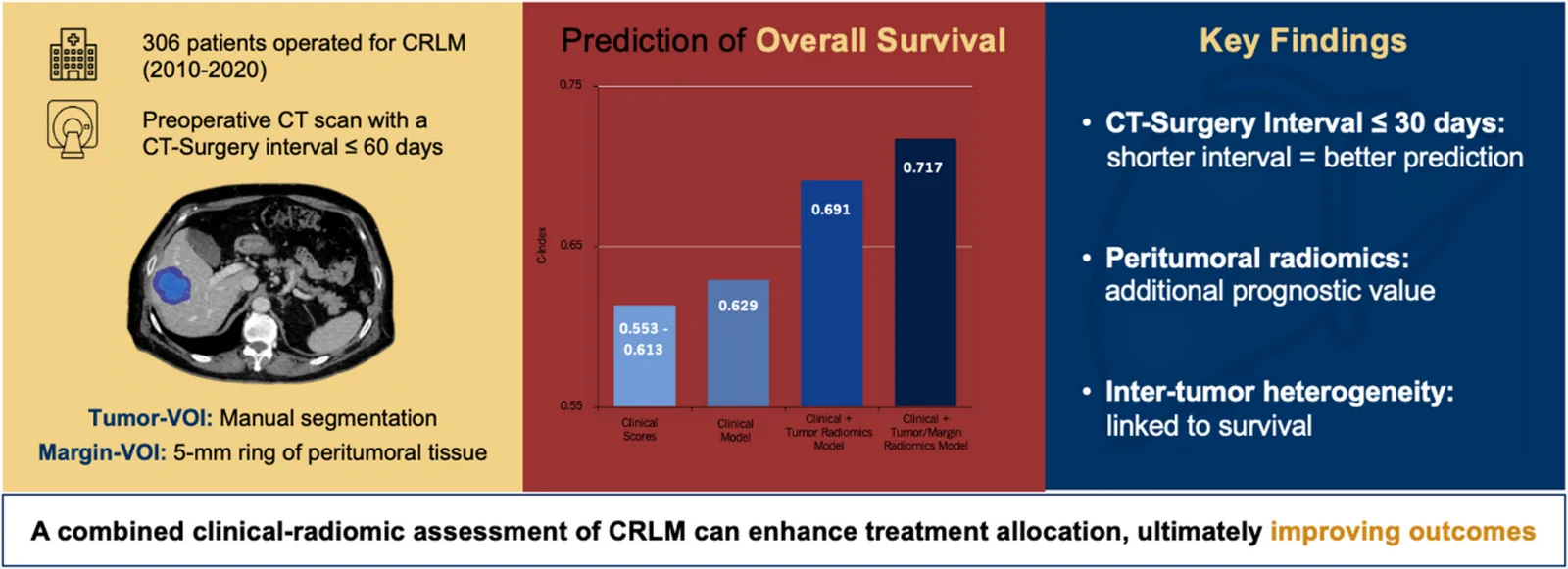

**Supplementary Information:**

The online version contains supplementary material available at https://doi.org/10.1007/s13304-026-02649-z.

## Introduction

Liver surgery with perioperative chemotherapy is a potentially curative treatment for colorectal liver metastases (CRLM) [1]. However, a subset of resectable patients does not benefit from surgery: after complete resection, early recurrence and early cancer-related death occur in 10–25% of cases, with higher rates observed in patients with a high tumor burden [2]. Several prognostic factors have been identified to refine patient selection for surgery, including characteristics of the primary tumor and metastases, pre-operative CEA levels, response to chemotherapy and genetic mutational status [3–5]. Consequently, numerous clinical prognostic scoring systems combining these factors have been proposed [6–9]. However, their performance at external validation is generally unsatisfactory, failing to provide an adequate prediction in up to one third of patients and potentially leading to an inadequate treatment allocation [10, 11]. This underscores the need for additional prognosticators to be considered.

Pathology data of CRLM and liver-tumor interface significantly influence disease progression and prognosis [12, 13], but conventional imaging exhibits suboptimal performance in their non-invasive evaluation [3, 14]. Many key biomarkers can only be defined on the resected surgical specimen, once the treatment strategy has already been established, highlighting a major unmet need in preoperative patient stratification. In this context, radiomics - an advanced imaging analysis technique based on computational algorithms to extract quantitative features from medical images - may enable a more comprehensive characterization of tumor biology beyond visual assessment alone [15]. In CRLM, radiomics has already shown promising results in the non-invasive assessment of pathological features and molecular profiles [16–19]. More recently, this approach has also been extended to the evaluation of the peritumoral microenvironment, which may provide additional prognostic information [20–22].

This study aims to identify radiomic features from both tumor and liver-tumor interface associated with the prognosis after liver resection for CRLM, to develop a comprehensive clinical-radiomic prognostic model and to compare its performance with that of a pure clinical model and established prognostic scores.

## Methods

The study was performed according to the declaration of Helsinki and its later amendments. The local review board approved the study protocol (83/20). The requirement for informed consent was waived due to the retrospective design of the study. The analyses had no impact on clinical decision-making, and a standard-of-care pathway was followed in every case. The study adhered to the CLEAR checklist designed for radiomic studies (Supplementary Table 1). The study is registered on Clinicaltrial.gov (NCT06779734).

We retrospectively considered all consecutive patients who underwent liver resection for CRLM between January 2010 and January 2020 at Humanitas Research Hospital, Milan, Italy. Inclusion criteria were: age ≥ 18 years; confirmation of diagnosis of CRLM at final pathology on the resected liver specimen; preoperative contrast-enhanced CT scan available for review with at least one detectable CRLM ≥ 10 mm. As previously reported [23], patients with tumor < 10 mm were excluded because the analysis of a 5-mm peritumoral volume was considered inadequate for tumor size. Exclusion criteria included: explorative laparotomy/laparoscopy without liver resection; other malignancies in the previous five years; preoperative imaging performed > 60 days before surgery and/or before the end of chemotherapy; inadequate CT scan, i.e. lack of an adequate portal phase, movement or high-density material artifacts affecting the analysis; prior loco-regional treatment, such as ablation. In patients receiving preoperative chemotherapy, post-treatment CT scan was considered.

The study focused on Overall Survival (OS) after resection, with the main goal of comparing the prognostic accuracy of a combined clinical-radiomic model against a pure clinical model or common scoring systems (Fong, GAME, and RAS mutation clinical risk scores [6, 9, 24], Supplementary Text 1). Radiomic features from both the tumor and the peritumoral tissue were included. Secondary objectives included: (1) assessing the impact of the interval between the CT scan and surgery (≤ vs. >30 days) on the prognostic accuracy of radiomic indices; (2) examining whether radiomic analysis of intra-patient inter-tumor heterogeneity improves survival prediction in patients with multiple CRLM; and (3) exploring the predictive performance of radiomic features extracted from pre-chemotherapy CT scans in patients who received preoperative systemic therapy.

All analyses were conducted on a per-patient basis. In patients with multiple CRLMs, the largest lesion was analysed, except for the heterogeneity analysis, in which up to five lesions per patient (the largest ones) were considered.

In a post-hoc sample size analysis, a total of 84 patients was adequate to demonstrate an increase in the C-index from 0.65 for the clinical model to 0.75 for the combined clinical–radiomic model, with 90% power, α = 0.05, and assuming a 50% event rate. With more than 200 patients under the same conditions, a difference in the C-index of 0.05 or greater was detectable.

### Image acquisition and analysis

CT scans performed at Humanitas Research Hospital, Milan, Italy were acquired according to a standardized protocol (Supplementary Text 2); specifically, the portal venous phase was acquired 75 s after contrast administration (Iomeron 300 mg/ml, Bracco Imaging, Italy).

Segmentation and radiomic analysis were performed with LifeX software (https://www.lifexsoft.org/) [25]. In patients with multiple lesions, up to five metastases (the largest ones ≥ 10 mm) were segmented. A Volume Of Interest (VOI) was manually drawn on the portal phase of the preoperative CT images to include the entire tumor (Tumor-VOI). Then, a VOI of peritumoral tissue was generated automatically by including a 5-mm thick rim around the Tumor-VOI (Margin-VOI) and manually corrected, if needed, to exclude non-liver parenchymal tissue.

Manual segmentations were performed independently by one of the two radiologists (AA and FF). To assess reproducibility, 10% of lesions were segmented by both, and inter-operator agreement was calculated using the Dice Similarity Coefficient and Intersection over Union (IoU).

Radiomic features were extracted automatically for each VOI using LifeX software (v7.5.0) according to the Image Biomarker Standardisation Initiative [26] (Supplementary Text 3). Consequently, a total of 110 features were analysed (morphological, intensity-based–HU, intensity-histogram, GLCM, GLRLM, NGTDM, GLSZM).

### Patient management

A multidisciplinary team planned the most adequate treatment in all cases. Patients amenable to a complete resection (R0/R1 surgery) were considered for surgery, independently of liver tumor burden. Perioperative chemotherapy was systematically considered, except for patients with very limited tumor burden and easy-to-resect CRLM. Patients with disease progression during chemotherapy were preferentially scheduled for a second-line treatment. Patients with unresectable diseases at diagnosis received systemic therapy and were considered for surgery when resectability was reached at restaging. Follow-up was performed every 3 months for the first two years after surgery, and every six months thereafter. It included CEA levels and abdominal ultrasonography, CT, or MRI; total colonoscopy was repeated annually.

### Statistical analyses

After completing the database, outliers were analyzed, verified, and corrected if needed. Missing clinical and genetic data were imputed using the Hyperimpute method [27]. Continuous variables were summarized with median and interquartile ranges or mean and range, while categorical variables with frequencies and proportions. The Kaplan-Meier method was used to estimate survival probabilities, and the log-rank test to compare different groups. OS was calculated from the date of surgery to the date of death or last follow-up. In all analyses, continuous variables were included as continuous predictors to maximize their contribution.

All collected clinical variables, selected for their potential prognostic value based on available literature [6, 9, 24, 28], were considered for the model (Supplementary Text 4). Given the potential high correlation of radiomic variables, a Spearman correlation analysis was performed with a threshold set at 0.8 to retain only non-redundant features, selecting the highest-order ones. This preliminary investigation yielded a dataset of 43 radiomic features for Tumor-VOI and 43 for Margin-VOI.

**Fig. 1:**
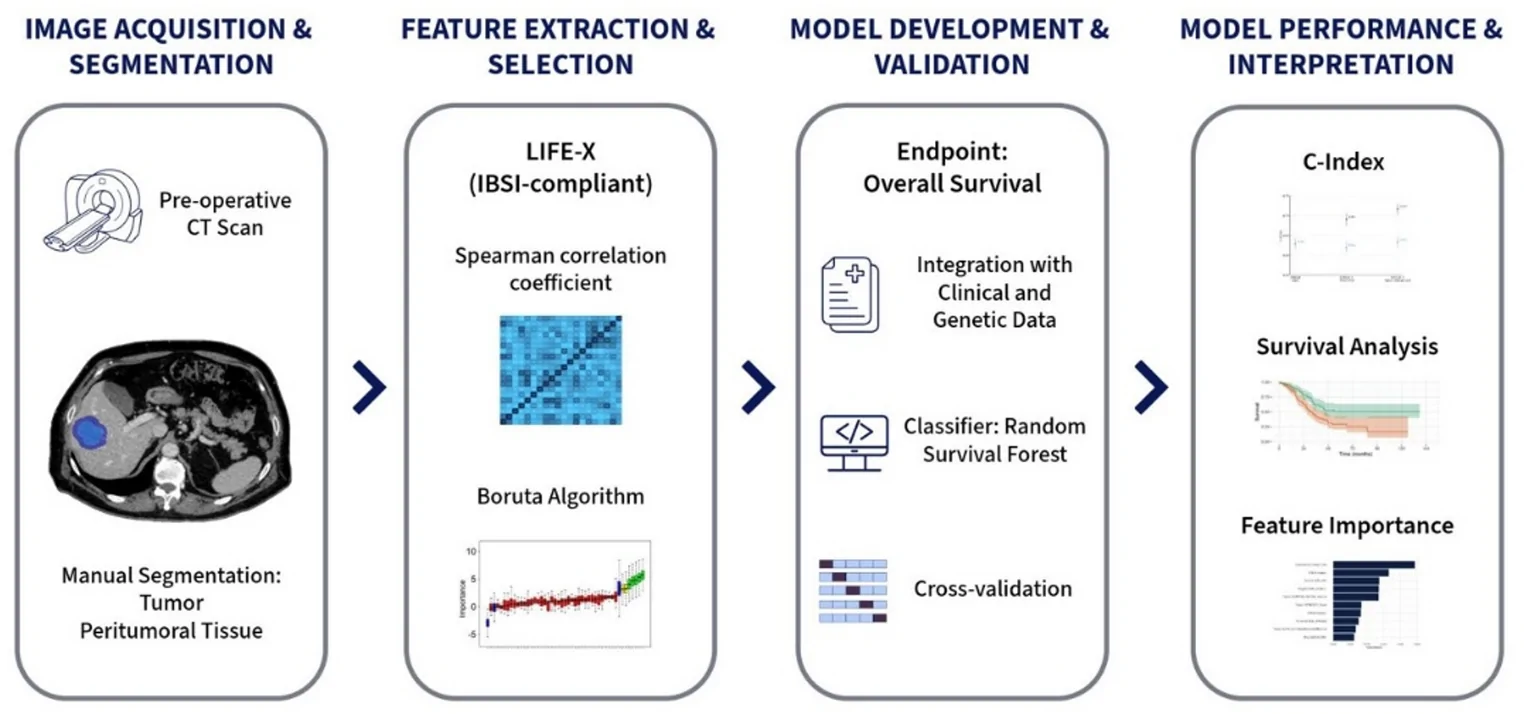
Schematic overview of the radiomics technical pipeline

Three predictive models were built by a two-step approach, including dimensionality reduction and modelling: (1) pure clinical model; (2) combined clinical-radiomic model with features from Tumor-VOI; (3) combined clinical-radiomic model with features from both Tumor- and Margin-VOIs. First, further feature selection was performed using the Boruta algorithm [29], which classifies variables according to their importance as prognosticators and selects those with importance higher than that of the random value. Secondly, a Random Survival Forest model was preferred to a standard Cox model to explore both linear and non-linear associations between predictors and outcome, as well as higher-order interactions among variables. Internal validation was then performed by computing the OOB C-index using 10-fold Cross Validation with 95% Confidence Intervals, and model interpretability was explored through feature importance plots. A univariate Cox analysis was performed to evaluate the performance of clinical scores. A summary of the technical workflow of the study is provided in Fig. 1.

For the intra-patient heterogeneity analysis, we adopted the methodology developed by Cavinato et al. [30]. Patients with multiple lesions were represented by dendrograms, with each leaf corresponding to a lesion and edges reflecting lesion similarity based on radiomic features. Euclidean pairwise distances between lesions were computed, followed by hierarchical clustering with average linkage to capture intra-patient lesion similarity. To group patients based on these dendrograms, we applied unsupervised clustering using the Tree-Edit distance [31], which quantifies differences between dendrograms. Multiple clustering algorithms were evaluated and compared to identify the most effective method. The resulting cluster labels were then used to stratify patients and quantify the predictive ability with respect to the outcome(s).

As an exploratory analysis, additional prognostic models were developed in the subgroup of patients who received preoperative chemotherapy and with available treatment-naïve CT scans, following the same pipeline described above. These aimed to evaluate the prognostic role of pre-treatment radiomic features and the potential influence of chemotherapy-related morphological changes on radiomic performance.

A *p* < 0.05 was considered significant for all tests. Stata 18.0 (StataCorp, College Station, TX, USA). and R (R Foundation for Statistical Computing, Vienna, Austria) were used.

## Results

**Fig. 2:**
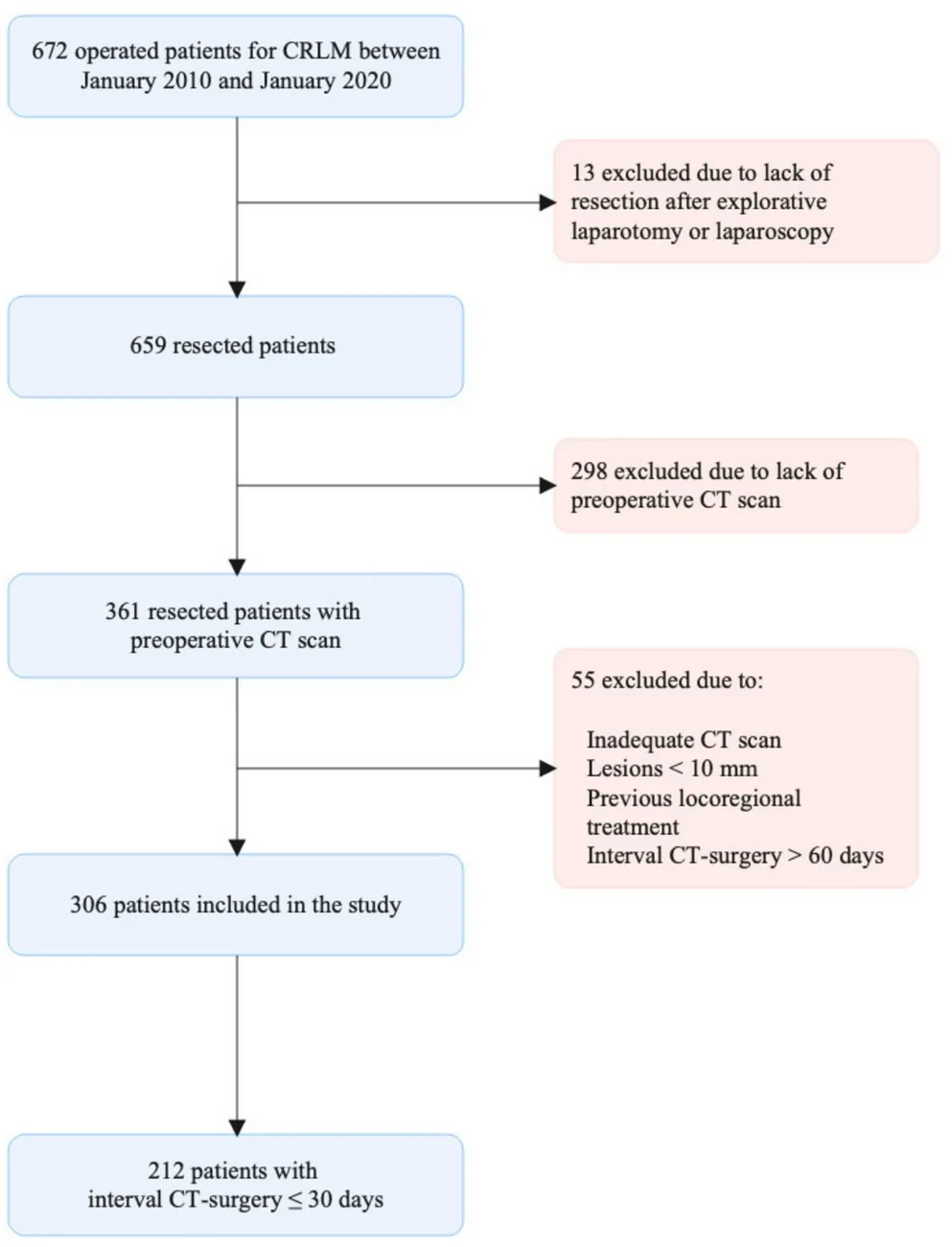
Flowchart of patient selection. CRLM, Colorectal liver metastases

Overall, 672 patients were operated for CRLM in the study period at the authors’ institution. Of these, 366 patients were excluded, mainly due to the absence of a preoperative CT scan available for radiomic analysis. A total of 306 patients were included and 767 metastases (up to 5 lesions ≥ 10 mm per patient) were analysed. The patient selection process is reported in Fig. 2.

The study population included 187 (61%) males and 119 (39%) females with a mean age in the whole cohort of 63 years (range 30–86). Half of the patients had primary tumor in the left colon (148 patients, 49%). CRLM were synchronous with the primary tumor in 181 (59%) patients, larger than 50 mm in 91 (30%), and multiple in 238 (78%). Genetic profile was available in 244 patients, and the proportion of KRAS, NRAS, and BRAF mutated patients were 28%, 3.7% and 1.2%, respectively. Preoperative chemotherapy was administered to about three-fourth of patients (*n* = 216), while adjuvant one in half of them (*n* = 140). Patients’ characteristics are summarized in Table 1.

**Table 1:** Patient characteristics

Patient characteristics	Whole series, per patient (*n* = 306)	Missing
Age, mean (range)	63 (30–86)	–
Sex (M)	187 (61%)	–
*Primary tumor*		
Right colon	69 (23%)	6
Left colon	148 (49%)	
Rectum	83 (28%)	
T3-T4	248 (86%)	19
N+	199 (69%)	18
*Liver metastases*		
Synchronous	181 (59%)	–
> 3 metastases	152 (50%)	–
Number, mean (range)	5 (1–37)	–
< 50 mm	215 (70%)	–
Diameter in mm, mean (range)	41 (10–155)	–
Extrahepatic disease	49 (16%)	–
RAS mutation	80 (33%)	62
BRAF mutation	3 (1%)	
Preoperative CEA < 200 ng/ml	275 (93%)	11
Preoperative chemotherapy	216 (71%)	–
Complete response	1 (0.5%)	–
Partial response	164 (76%)	
Stable disease	35 (16%)	
Disease progression	16 (7.5%)	
Postoperative chemotherapy	140 (46%)	7
Severe morbidity	22 (7%)	–
Infective morbidity	32 (10%)	–
Death at 90 days	5 (1.6%)	–
Resection margin > 1 mm	190 (62%)	–
*Clinical scores*		
Fong score		
Low-risk (score 0–2)	203 (73%)	28
High-risk (score 3–5)	75 (27%)	
*RAS mutation clinical risk score*		
Score 0	33 (12%)	31
Score 1	110 (40%)	
Score 2	114 (41%)	
Score 3	18 (7%)	
*GAME score*		
Low-risk (score 0–1)	33 (12%)	39
Intermediate-risk (score 2–3)	157 (59%)	
High-risk (score 4+)	77 (29%)	

After a mean follow up of 34 months (range 1-137 months; 44 months in alive patients), the three- and five-year OS rates were 53.7% and 40.9%, respectively (Supplementary Fig. 1). Of the 306 included patients, 33 (11%) survived more than 5 years after hepatectomy (18 of whom experienced recurrence), 144 died of disease within 5 years of follow-up, and 129 were alive at the last contact with a follow-up shorter than 5 years (median follow-up, 34.6 months; 93 with recurrence).

Of the 306 included patients, 212 (69%) had an interval between the CT scan and surgery ≤ 30 days. All segmentations were successfully completed. In the 10% sample of patients with double manual segmentations, the inter-operator agreement was excellent (median Dice coefficient = 0.899, IoU = 0.817).

### Survival prediction: clinical model

**Fig. 3:**
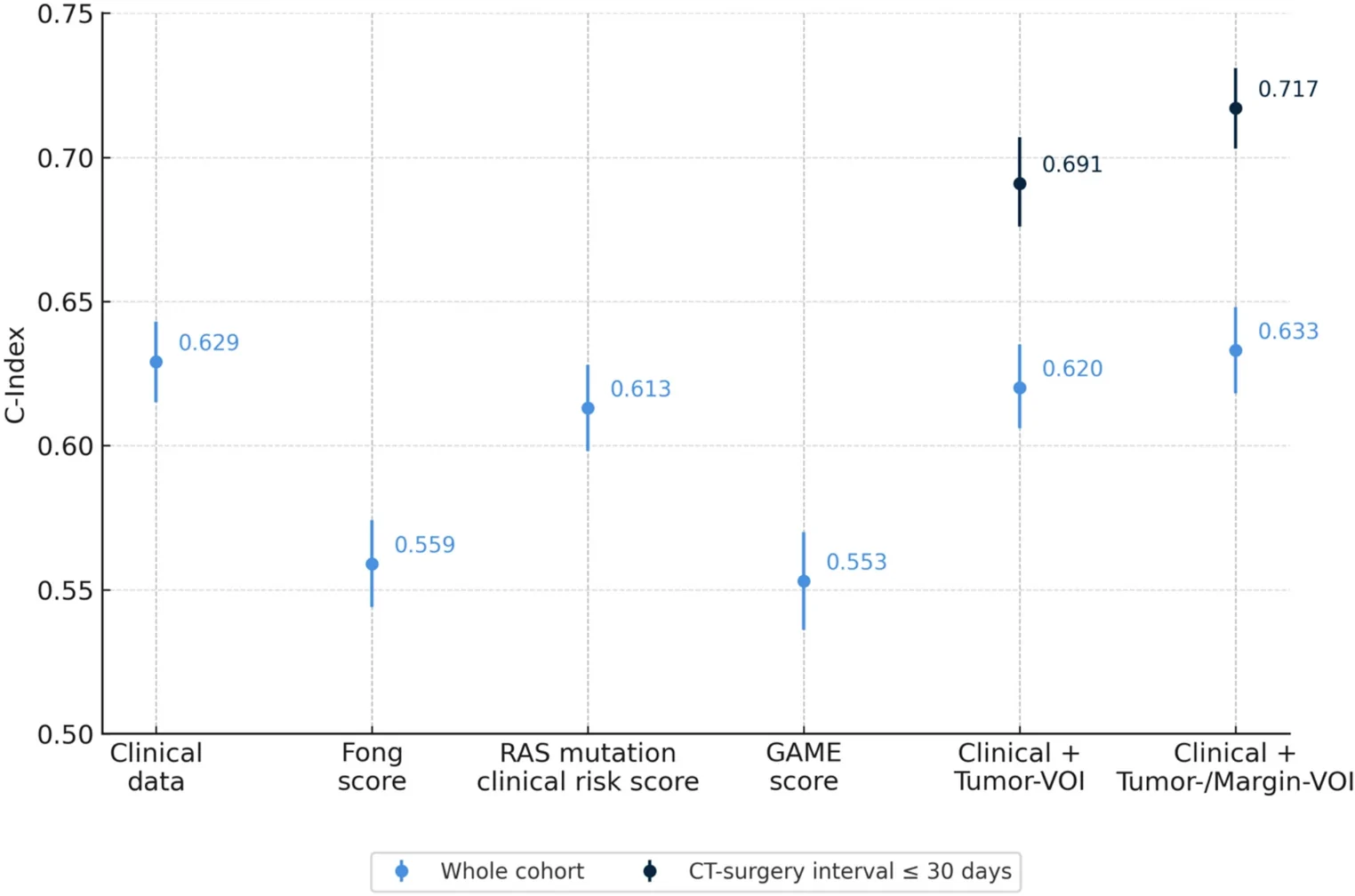
Performance of the models and the prognostic scores (C-Index and 95% CI) in the whole cohort (light blue) and in the subgroup of 212 patients with a CT-surgery interval ≤ 30 days (blue)

**Fig. 4:**
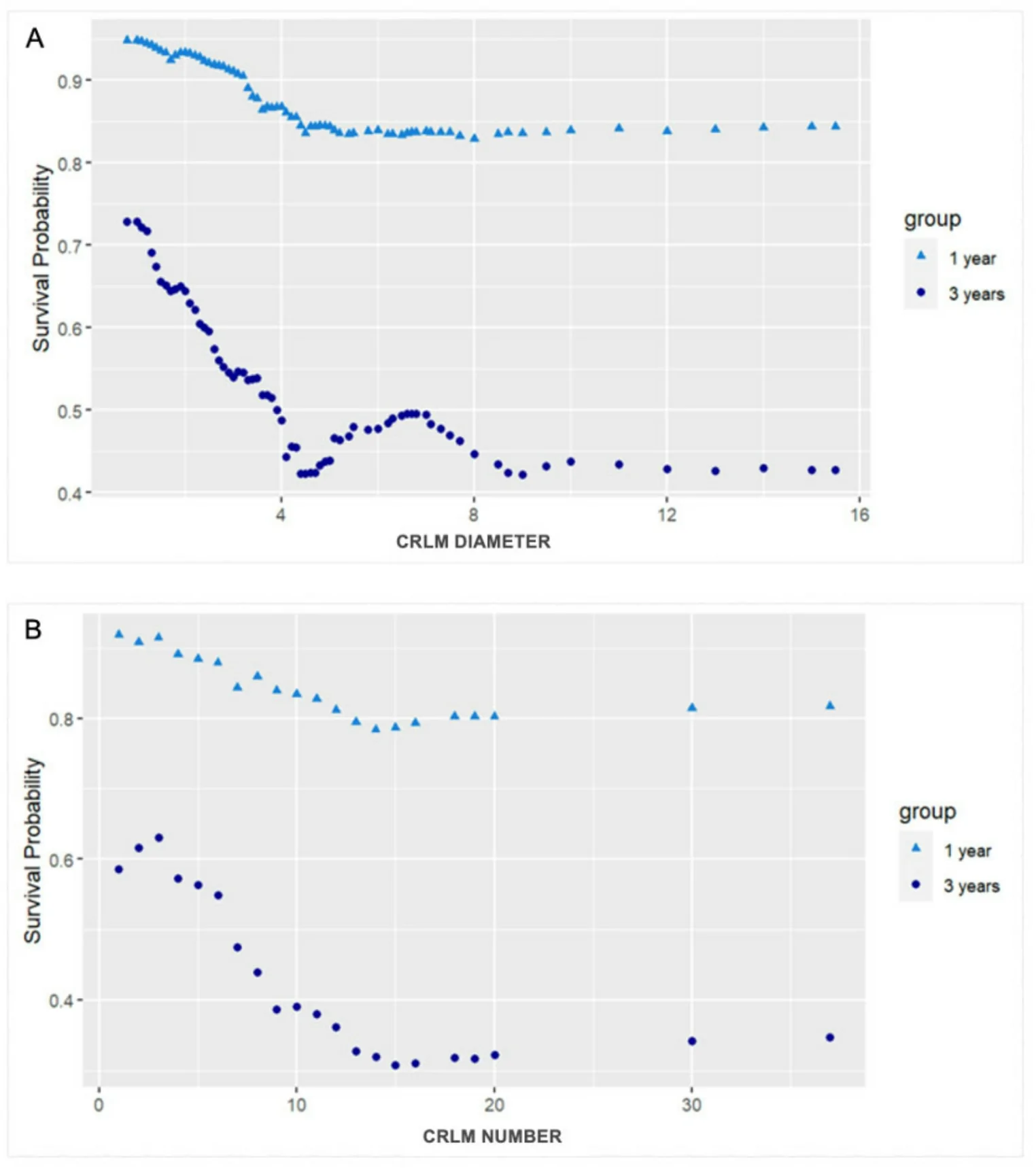
Partial Effect plots of number (**A)** and diameter (**B)** of liver metastases on survival probability. CRLM, Colorectal liver metastases

The pure clinical model retained three variables: number and size of CRLM and presence of extrahepatic disease. At internal validation, it achieved a C-index of 0.629 (95%CI:0.615–0.643). The model is reported in Supplementary Fig. 2, while its performance in Fig. 3; Table 2. Genetic data did not contribute to outcome prediction. Tumor number and size showed a non-linear association with OS that progressively decreased up to 15 metastases and 40 mm, respectively, but remained stable beyond these thresholds (Fig. 4). Prognostic Fong, RAS Mutation Clinical Risk and GAME scores had low performance with C-indexes of 0.559, 0.613, and 0.553, respectively (Fig. 3; Table 2).

**Table 2:** Comparison of the performance of the clinical scores, pure clinical, and combined models in the whole cohort and in the subgroup of 212 patients with a CT-surgery interval ≤ 30 days at internal validation

Prognostic model	OOB C-index (95% CI)	
	Whole cohort (306 patients)	Delta date ≤ 30 day (212 patients)
Clinical	0.629 [0.615; 0.643]	
Fong score	0.559 [0.544; 0.573]	
RAS mutation clinical risk score	0.613 [0.598; 0.628]	
GAME score	0.553 [0.536; 0.570]	
Clinical data + tumor radiomics	0.620 [0.606; 0.635]	0.691 [0.676; 0.707]
Clinical data + tumor radiomics + margin radiomics	0.633 [0.618; 0.648]	0.717 [0.703; 0.731]

### Survival prediction: clinical-radiomic model

The contribution of radiomics to OS prediction was limited in the entire cohort (C-index = 0.633, 95%CI:0.618–0.648) but increased in the subgroup of patients who underwent CT within 30 days prior to surgery. In this subgroup, at internal validation, the model combining clinical data with radiomic features from the Tumor VOI achieved a C-index of 0.691 (95%CI:0.676–0.707), increasing to 0.717 (95%CI:0.703–0.731) when radiomic features from Margin VOI were added. These combined models outperformed both the purely clinical model and standard prognostic scores.

**Fig. 5:**
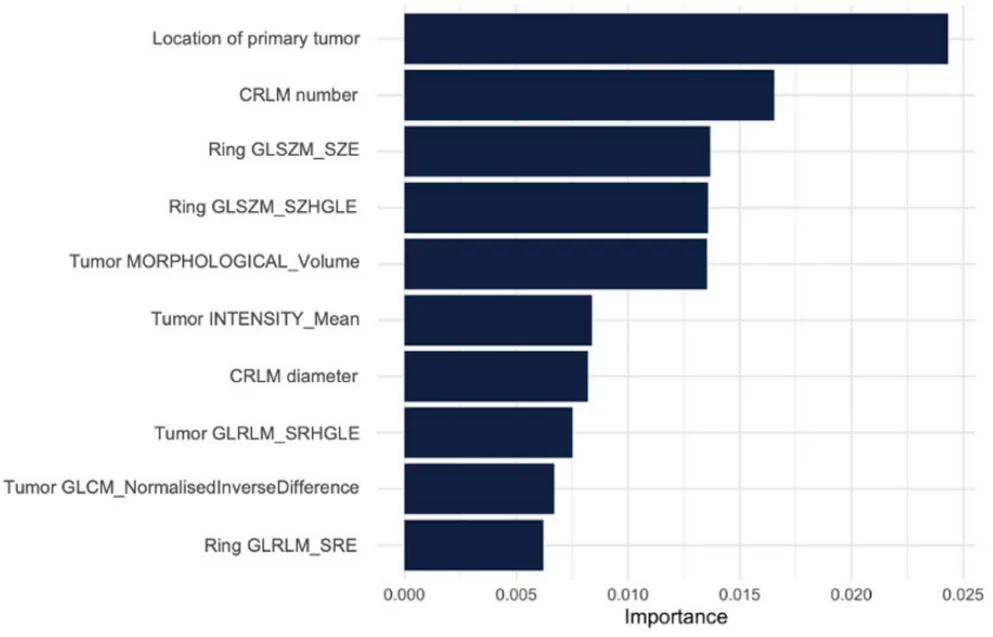
Feature importance plot of the combined model with clinical data and radiomic features from the tumor and the margin in the subgroup of 212 patients with a CT-surgery interval ≤ 30 days, CRLM, Colorectal liver metastases

The final model retained ten variables: three clinical (primary tumor sidedness, number and size of metastases) and seven radiomics (Fig. 5). Partial Effect plots and Kaplan-Meier curves for the five most significant variables of the model are presented in Supplementary Fig. 3.

Models and their performance are summarized in Figs. 3 and 5; Table 2 and Supplementary Fig. 2.

### Analyses of intra-patient lesion heterogeneity

The optimal clustering method was hierarchical clustering employing Ward linkage, Canberra distance, Tumor-VOI radiomics only, and including patients with a minimum of three lesions. This approach stratified patients into three clusters based on the number and the heterogeneity of the lesions.

**Fig. 6:**
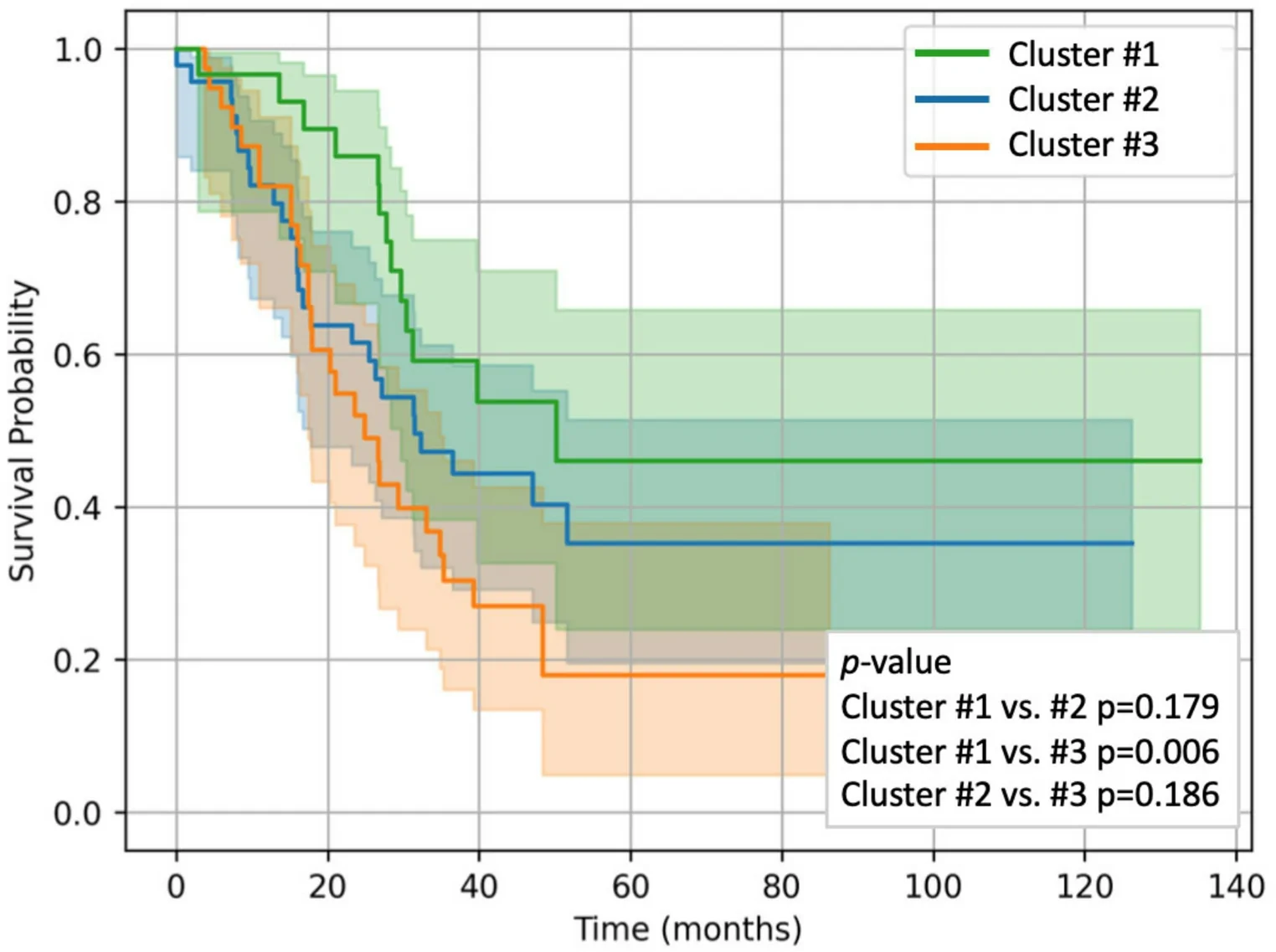
Kaplan-Meier curve for each cluster identified by hierarchical clustering (stdCoreCa3): Cluster #1 - low number of metastases and high inter-tumor heterogeneity; Cluster #2 - high number of metastases and low heterogeneity; Cluster #3 - high number of metastases and high heterogeneity

Although these clusters did not correspond to neat differences in clinical, genetic, or radiomic characteristics, they demonstrated significant associations with OS. Specifically, Cluster #1, characterized by a low number of metastases and high inter-tumor heterogeneity, was associated with the best OS. Cluster #2, comprising patients with a high number of metastases and low heterogeneity, exhibited intermediate OS. Cluster #3, with both a high number of metastases and high heterogeneity, showed the poorest survival outcomes (*p* = 0.006 vs. Cluster #1, Fig. 6). However, incorporating these clusters into combined predictive models did not improve the overall prognostic performance (Supplementary Fig. 4).

### Exploratory analysis on the pre-chemotherapy CT scans

Among the 216 patients who received preoperative chemotherapy, pre-chemotherapy CT scans were available for 102.  The performance of the three predictive models (pure clinical model and combined models using features from the Tumor-VOI alone or in combination with Margin-VOIs) did not exceed that of models based on pre-surgical CT scans (C-index range: 0.564–0.617).

## Discussion

The present study demonstrated a clear contribution of radiomics to overall survival prediction in patients undergoing complete resection for colorectal liver metastases. Prognostic models and scores based on morphological and genetic variables showed limited performance (C-index < 0.65), while radiomic features extracted from both tumor and peritumoral tissue improved predictive accuracy. The time interval between CT imaging and surgery was crucial: radiomics maximized its contribution when imaging was performed within a month prior to surgery. Analysis of inter-lesion heterogeneity, although associated with survival, had not significant impact on model performance.

An accurate prediction of survival in patients affected by CRLM is key to a precision-based multidisciplinary approach, enabling assessment of the expected benefit from resection, weighting alternative treatments, and distinguishing between candidates for upfront surgery and those requiring neoadjuvant treatments [1, 3, 32–37]. Over the past three decades, several prognostic scores have been proposed, initially focused on morphological factors and then integrating biological and genetic data [6, 9, 24]. However, their performance at external validation remained unsatisfactory with C-indexes ranging from 0.57 to 0.63 [6, 7, 9–11, 38], in line with our results (C-indexes < 0.65), indicating that over one-third of predictions were incorrect. The number and size of CRLM are strong predictors, both in the literature and in this analysis, although different cut-offs have been proposed, mainly based on a priori stratification [39, 40]. In our study, number and size were included as continuous variables, identifying a progressively decreasing prognosis up to 15 metastases and 40 mm in size, with a plateau for higher values. Moreover, tumor sidedness was confirmed as a prognostic factor, with right-sided tumors associated with a worse prognosis. This finding has been increasingly recognized, but only recently incorporated into prognostic scores [8].

The contribution of radiomics to survival prediction after CRLM resection has been previously reported [18, 41–43]. Tumor textural features and patient clustering based on their radiomic characteristics from preoperative CT scans have been associated with OS, recurrence-free survival, and early recurrence risk [41, 43–45]. CT-based radiomics also allowed for an accurate, non-invasive estimation of the pathological tumor response to chemotherapy, which is one of the strongest prognostic factors [23, 46]. Our model not only confirmed these findings but also demonstrated that the analysis of peritumoral tissue further improved prediction. These features can capture clinically relevant characteristics at the liver-tumor interface, such as tumor growth pattern, immune infiltration, and micrometastases [12, 13, 47], which are not assessable using standard preoperative imaging [3]. Fiz et al. demonstrated that tissue surrounding CRLM, although similar to the normal liver parenchyma on imaging, exhibits a radiomic profile more similar to the tumor itself, that gradually normalizes within the first perilesional centimeter [20]. Preliminary studies have also reported associations between textural indices and pathological characteristics of the liver–tumor interface [21, 22, 48], supporting the biological rationale behind the prognostic value of Margin-VOI features. A broader approach has also proven effective: radiomic features of the entire non-tumoral liver parenchyma have been associated with both the risk of CRLM recurrence after complete resection [43] and the appearance of metachronous metastases in patients with non-metastatic colorectal cancer [49]. Although heterogeneous methodologies are pursued, there is consistent evidence supporting the value of extending radiomic analyses beyond the tumor itself.

A further finding deserves attention: the impact of the interval between CT and surgery on radiomic performance. Even if all radiomics studies included patients with an “adequate” interval between imaging and surgery, definition varied, ranging from 30 to 90 days. In our cohort, radiomics demonstrated the strongest prognostic value when the CT-surgery interval was ≤ 30 days. Even though this cut-off could sound logical, consistent with Item #7 of the METRICS checklist [50], it has not been formally validated in the literature. The observation that radiomic characteristics may evolve significantly over such a short time frame might seem unexpected, but clinical evidence supports it. In patients with CRLM receiving preoperative systemic therapy, tumor reactivation occurs in 15–20% of cases within the 4–6 weeks interval between the end of chemotherapy and surgery, with morphological changes that negatively impact prognosis [51]. CT scans performed at the end of chemotherapy may fail to capture these changes, reinforcing the idea that imaging performed closer to surgery is needed to maximize the benefit of radiomic assessment.

Tumor heterogeneity is a major area of interest and a key focus of current research, as the most aggressive cellular clones (intra-lesional heterogeneity) and metastatic lesions (intra-patient inter-lesional heterogeneity) are increasingly recognized as determinants of outcomes [52]. Radiomics holds potential for assessing such heterogeneity, but only exploratory analyses have been reported testing different approaches [43, 45, 53]. A recent study by Marzi et al. evaluated the mean radiomic features extracted from the five largest CRLMs but found no significant impact on outcome prediction [43]. In the present analysis, which included three-fourths of patients with multiple lesions, an unsupervised hierarchical clustering approach was adopted, and an interesting finding emerged: patients with numerous metastases showed better survival when the lesions were homogeneous rather than heterogeneous. However, incorporating heterogeneity into the predictive models did not improve overall performance. This may reflect the limited heterogeneity among metastases within the same organ. Analyzing different disease sites could lead to different results. Further analyses focused on this topic are needed.

The present findings are clinically relevant for several reasons. They highlight the importance of incorporating radiomics into clinical practice and prognostication for CRLM. Notably, these results were obtained in a real-world setting, using routine CT scans without dedicated imaging protocols, supporting the feasibility of the approach. Without replacing standard clinical predictors, the combined clinical-radiomic model provided a more accurate prognostic assessment, with the potential to significantly influence treatment strategies. Furthermore, we identified key strategies to optimize the impact of radiomics, including the ideal timing of imaging (within 30 days before surgery) and the importance of extending analysis to the liver–tumor interface.

The present study has some limitations. First, it is a retrospective, single-center analysis across a ten-year period. Although clinical and radiological standards have remained largely consistent, a prospective, multicenter study with a standardized CT acquisition protocol and planned external validation is needed to confirm these findings. Second, recurrence-free survival was not analyzed. Even if including it could have provided a more complete overview of surgical outcomes, the resulting volume of data would have been difficult to summarize and interpret. Furthermore, we consider OS the most robust endpoint, while recurrence events need to be interpreted in light of their site and re-treatability, as both factors affect their clinical relevance [54–56]. Third, we did not investigate the correlation between radiomic features and pathological data, which is important to enhance their interpretability and clinical acceptability. Finally, radiomics still faces challenges regarding clinical applicability [57]. Manual segmentation introduces operator-dependent variability (despite high concordance among operators in our cohort) and is time-consuming. Radiomics-based prognostic scores are not easy to compute and lack standardized cut-off values for the extracted features. Ongoing technological advancements and integration of artificial intelligence into clinical workflows are expected to address these limitations.

In conclusion, radiomic features derived from the portal phase of preoperative CT scans improve the prediction of survival after liver resection for colorectal metastases, particularly when the imaging-to-surgery interval does not exceed one month and the peritumoral tissue is included in the analysis. Integrating radiomics into clinical practice holds strong potential for enhancing decision-making and personalizing treatment strategies, ultimately improving patient outcomes.

## Supplementary Information

Below is the link to the electronic supplementary material.

## Data Availability

Data are available from the corresponding author upon reasonable request.

## Abbreviations

CRLM: Colorectal liver metastases
IoU: Intersection over union
OS: Overall survival
VOI: Volume of interest

## Data Availability

Data are available from the corresponding author upon reasonable request.
