# Correction: CT-based radiomics improves survival prediction in colorectal liver metastases: beyond clinical scores

**DOI:** 10.1007/s13304-026-02778-5

**Published:** 2026-07-24

**Authors:** Angela Ammirabile, Gilda Matteucci, Francesco Fiz, Elisa Ragaini, Sofia Moroni, Ezio Lanza, Lara Cavinato, Jacopo Galvanin, Chiara Masci, Guido Costa, Andrea Laghi, Guido Torzilli, Francesca Ieva, Luca Viganò

**Affiliations:** 1 Department of Biomedical Sciences, Humanitas University, Via Rita Levi Montalcini 4, Pieve Emanuele, Milan, 20072 Italy; 2 Department of Diagnostic and Interventional Radiology, IRCCS Humanitas Research Hospital, Milan, Italy; 3 MOX Laboratory, Department of Mathematics, Politecnico di Milano, Milan, Italy; 4 Nuclear Medicine Unit, Department of Diagnostic Imaging, Ente Ospedaliero Ospedali Galliera, Genoa, Italy; 5 Division of Hepatobiliary and General Surgery, Department of Surgery, IRCCS Humanitas Research Hospital, Milan, Italy; 6 Hepatobiliary Unit, Department of Minimally Invasive General and Oncologic Surgery, Humanitas Gavazzeni University Hospital, Viale M. Gavazzeni 21, Bergamo, 24125 Italy

**Correction to: Updates in Surgery** **https://doi.org/10.1007/s13304-026-02649-z**

In the original version of this article, the all author names were incorrect order. Now the name order was displayed correctly in all versions at the time of publication.

The correct author order list as follows:

Angela Ammirabile^1,2^ · Gilda Matteucci^3^ · Francesco Fiz^4^ · Elisa Ragaini^1^ · Sofia Moroni^3^ · EzioLanza^1,2^ · Lara Cavinato^3^ · Jacopo Galvanin^5^ · Chiara Masci^3^ · Guido Costa^1,5^ · Andrea Laghi^1,2^ · Guido Torzilli^1,5^ · Francesca Ieva^3^ · Luca Viganò^1,6^

The wrong author order list as follows:

Angela Ammirabile^1,2^ · Gilda Matteucci^3^ · Francesco Fiz^4^ · Elisa Ragaini^1^ · Sofia Moroni^3^ · Ezio Lanza^1,2^· Lara Cavinato^3^ · Jacopo Galvanin^5^ · Chiara Masci^3^ · Guido Costa^1,5^ · Andrea Laghi^1,2^ · Luca Viganò^1,6^ Francesca Ieva^3^ · Guido Torzilli^1,5^

The original article has been corrected.

