# Supplementary material for: CT-based radiomics improves survival prediction in colorectal liver metastases: beyond clinical scores
Source: Updates Surg. 2026 Jun 17;78(5):1889–900. doi: 10.1007/s13304-026-02649-z (PMC13538214; doi:10.1007/s13304-026-02649-z)
Supplement: Supplementary file 1 [file 13304_2026_2649_MOESM1_ESM.pdf]

CT Radiomics Improves Survival Prediction in Colorectal Liver Metastases: Beyond Clinical Scores

Angela Ammirabile, Gilda Matteucci, Francesco Fiz, Elisa Ragaini, Sofia Moroni, Ezio Lanza, Lara Cavinato, Jacopo Galvanin, Chiara Masci, Guido Costa, Andrea Laghi, Guido Torzilli, Francesca Ieva, Luca Viganò

Supplementary Material

Supplementary Text 1. Details of the evaluated scoring systems

- **FONG Score** (+1 point per item; low-risk 0-2 points, high-risk 3-5 points): node-positive primary, disease-free interval from primary to metastases <12 months, number of hepatic tumors >1, largest hepatic tumor >5 cm, carcinoembryonic antigen level >200 ng/ml.

- **GAME Score** (low-risk 0-1 points, intermediate-risk 2-3 points, high-risk 4+ points): KRAS-mutated tumors (1 point), carcinoembryonic antigen level ≥ 20 ng/ml (1 point), node-positive primary (1 point), Tumour Burden Score 3-8 (1 point) or ≥ 9 (2 points), extrahepatic disease (2 points).

- **RAS Mutation Clinical Risk Score** (+1 point per item): node-positive primary, RAS-mutated tumors, largest hepatic tumor >5 cm.

Supplementary Text 2. Image acquisition protocol

The analysed CT scans were acquired according to a standardized protocol: breath-holding acquisitions, including a pre-contrast basal phase, a bolus-triggered arterial phase, a portal phase (75 seconds after contrast administration) and a late phase (3 minutes after contrast administration). The iodine contrast media (Iomeron 300 mg/ml, Bracco Imaging, Italy) injected in bolus, followed by a 30-ml 0.9% saline solution flush. To time arterial phase acquisitions, bolus tracking over the abdominal aorta near the celiac trunk was used, with a threshold of 120 Hounsfield units. CT images were acquired on three different devices (Philips and GE Healthcare) with a similar set-up in terms of detector collimation (0.625 mm), current (280–400 mA), tension (120–140 kV) and pitch (0.975). We also used automatic exposure control based on the X-ray attenuation on scout images.

Supplementary Text 3. Radiomic feature extraction

Radiomic features were extracted using the IBSI-compliant software LIFEx v7.5. Unless otherwise specified, all parameters were kept at the software default. Gray-level discretization was performed with a fixed bin width of 10 within an absolute intensity range of −1000 to 3000 HU, with textural features computed in 3D. No image resampling was applied. Features were derived exclusively from the original portal venous phase CT images (Tumor-VOI and 5-mm peritumoral Margin-VOI), without any additional filtering or transformed image types.

Supplementary Text 4. Analysed clinical variables

The analysed clinical variables included the following parameters:

- Demographic data - sex and age
- Primary tumor - sidedness, T status, and N status
- Liver metastases characteristics - synchronous/metachronous, number, and size at surgery
- Concomitant extra-hepatic disease
- Mutational status of KRAS, NRAS, and BRAF
- Pre-operative CEA value
- Pre-operative chemotherapy data - administration, regimen, number of lines and cycles, and radiological response (RECIST v1.1)
- CT-surgery interval
- Post-operative data – 90-day mortality, infective/severe morbidity, resection margins (R0/R1), adjuvant chemotherapy.

Supplementary Figure 1. Kaplan–Meier curve for overall survival

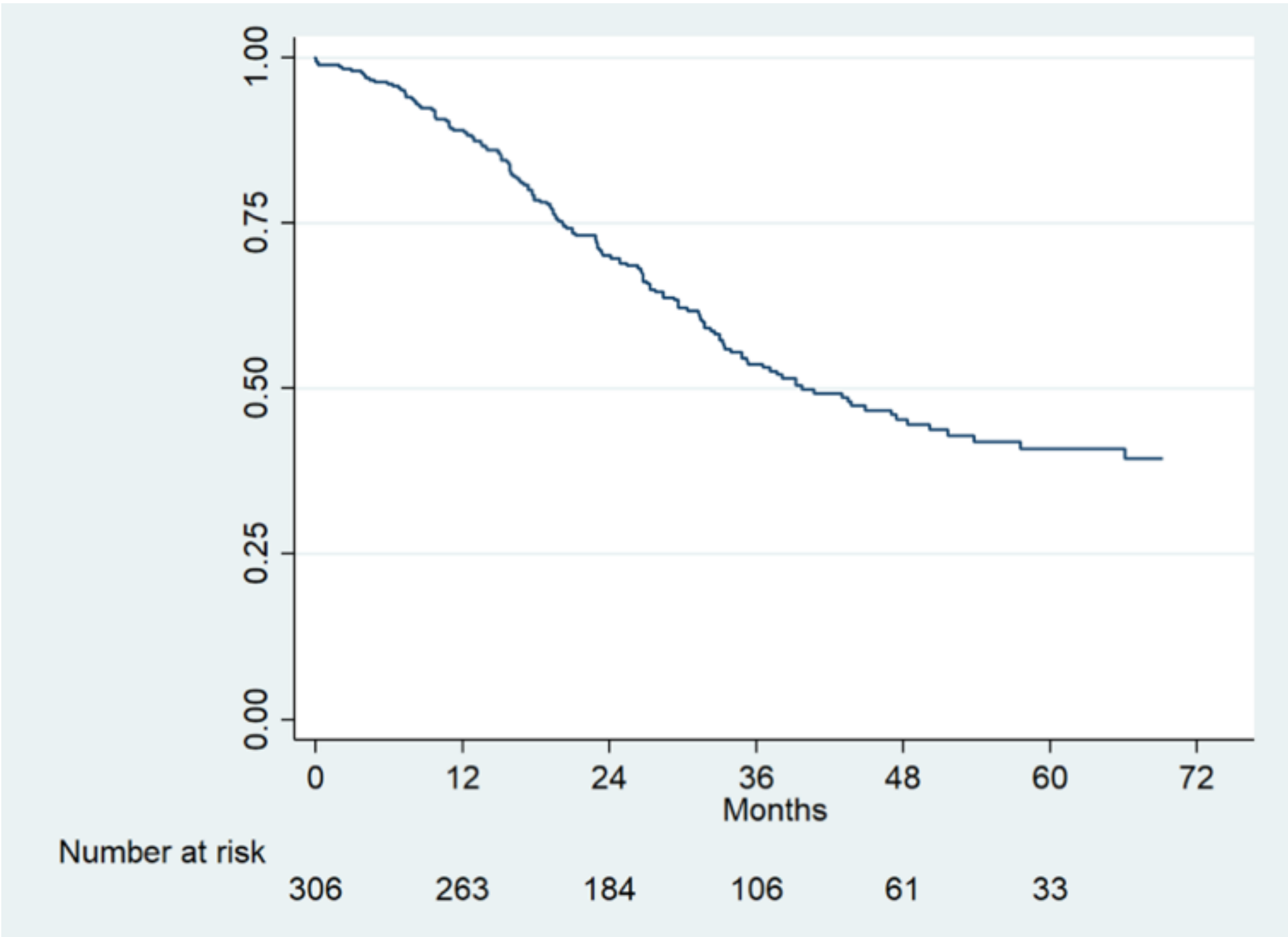

Supplementary Figure 2. Feature importance plots of the developed models in the whole cohort (A - pure clinical, B - combined clinical and Tumor-radiomics, C - combined clinical and Tumor/Margin-radiomics) and in the subgroup of patients with a CT-surgery interval ≤ 30 days (D - combined clinical and Tumor-radiomics)

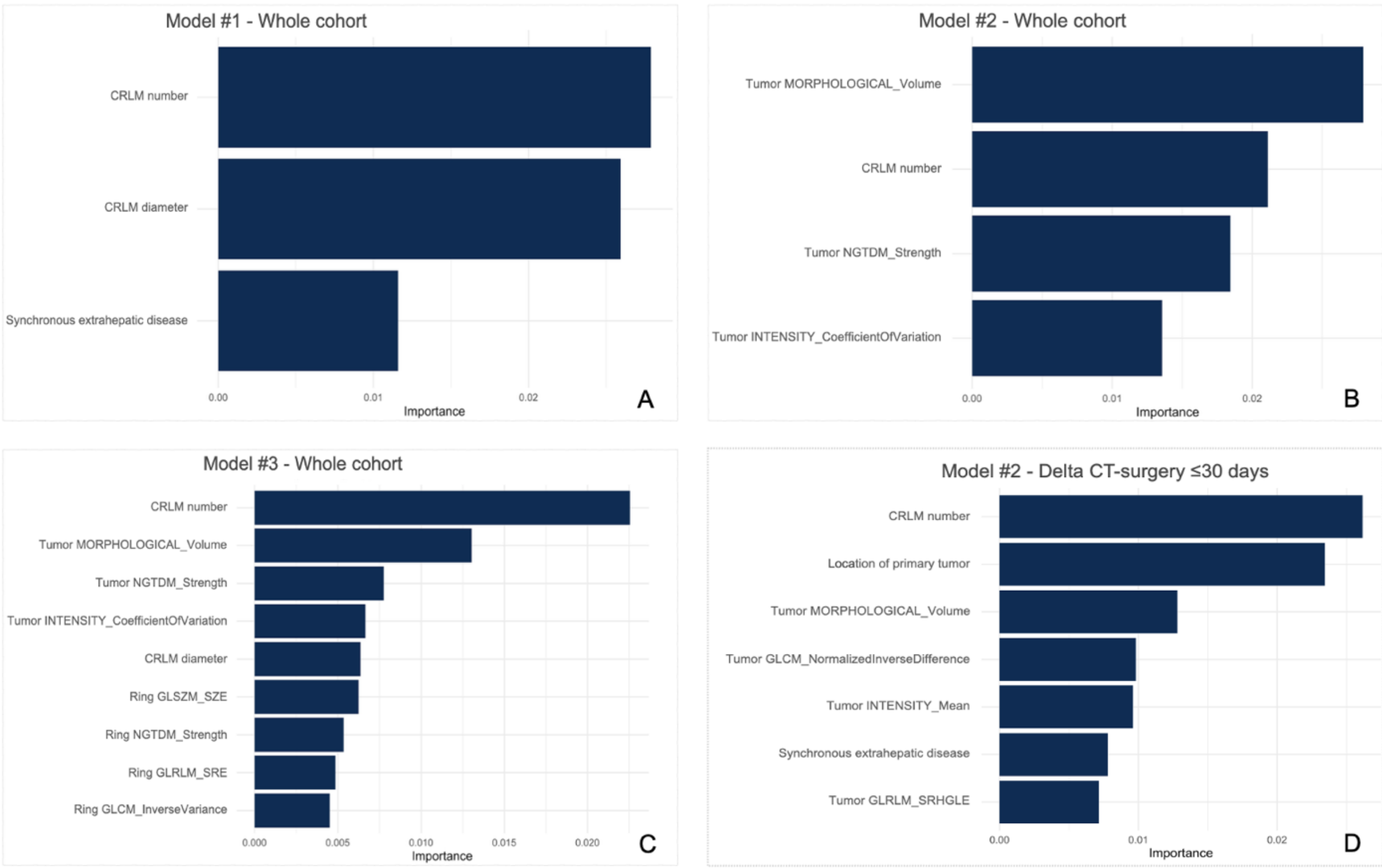

Supplementary Figure 3. Partial Effect plot (A, C, E, G, I) and Kaplan-Meyer (B, D, F, H, J) of the five most important variables of Model #3 (combined clinical and Tumor/Margin-radiomics) in the subgroup of patients with the CT-surgery interval ≤ 30 days. The median split values for the Kaplan-Meyer curves were as follows: 3 lesions for CRLM number; 0.597 for Ring GLSZM_SZE; 7314.81 for Ring GLSZM_SZHGLE; 10678.23 mm3 for Tumor MORPHOLOGICAL_Volume

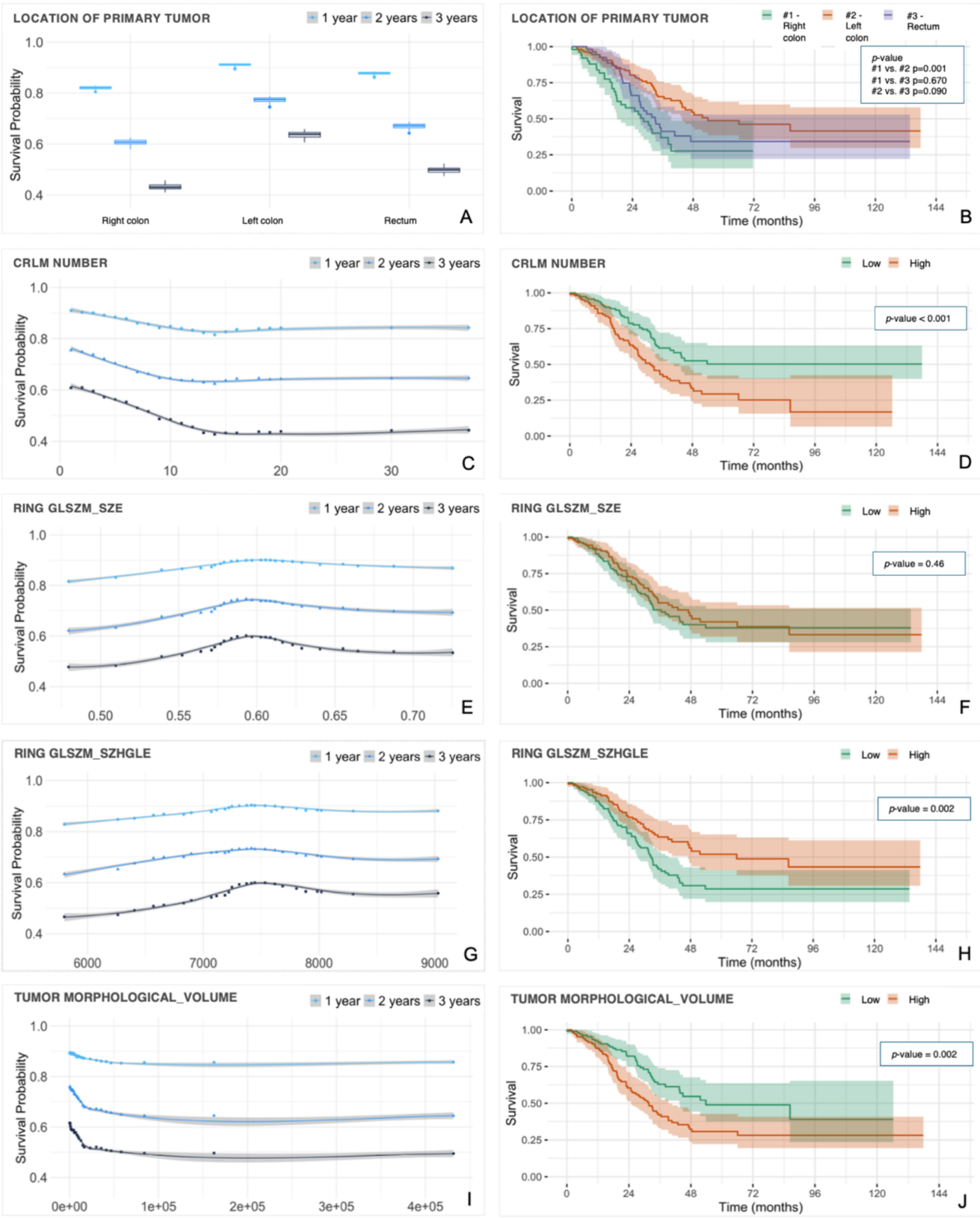

Supplementary Figure 4. Performance of the models (c-index and 95% CI) including a heterogeneity-based stratification variable (stdCoreCa3) in the whole cohort (light blue) and in the subgroup of 212 patients with a CT-surgery interval ≤ 30 days (blue)

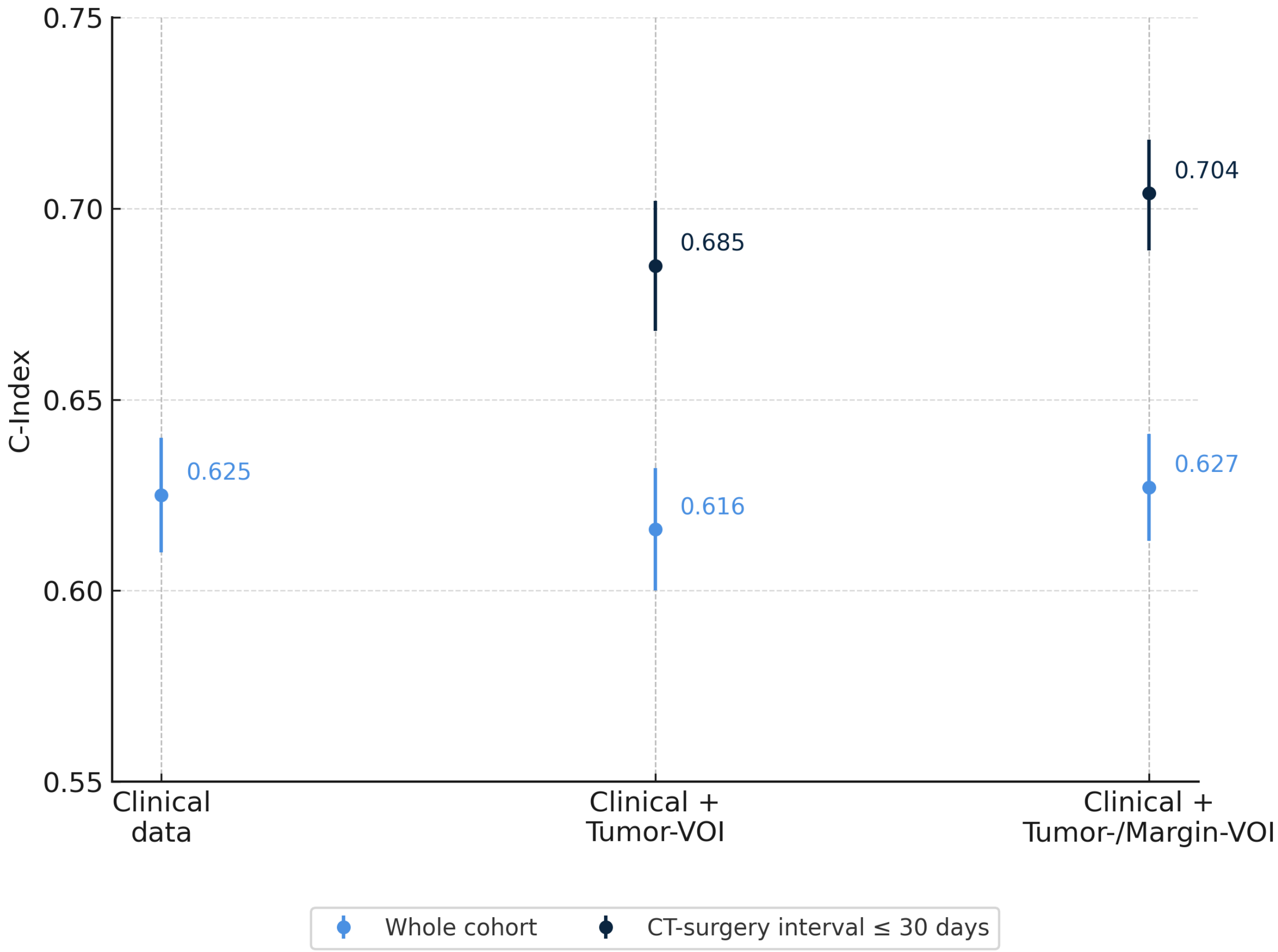

Supplementary Table 1. CLEAR Checklist

Section	No.	Item	Yes	No	n/a	Page
**Title**						
	1	Relevant title, specifying the radiomic methodology	X	☐	☐	1
**Abstract**						
	2	Structured summary with relevant information	X	☐	☐	2-3
**Keywords**						
	3	Relevant keywords for radiomics	X	☐	☐	3
**Introduction**						
	4	Scientific or clinical background	X	☐	☐	4
	5	Rationale for using a radiomic approach	X	☐	☐	4
	6	Study objective(s)	X	☐	☐	4
**Method**						
Study Design	7	Adherence to guidelines or checklists (e.g., CLEAR checklist)	X	☐	☐	5
	8	Ethical details (e.g., approval, consent, data protection)	X	☐	☐	5
	9	Sample size calculation	X	☐	☐	6
	10	Study nature (e.g., retrospective, prospective)	X	☐	☐	5
	11	Eligibility criteria	X	☐	☐	5
	12	Flowchart for technical pipeline	X	☐	☐	Fig 1
Data	13	Data source (e.g., private, public)	X	☐	☐	5
	14	Data overlap	☐	X	☐	-
	15	Data split methodology	☐	☐	X	-
	16	Imaging protocol (i.e., image acquisition and processing)	X	☐	☐	S.Text 2
	17	Definition of non-radiomic predictor variables	X	☐	☐	S.Text 4
	18	Definition of the reference standard (i.e., outcome variable)	X	☐	☐	5, 7
Segmentation	19	Segmentation strategy	X	☐	☐	6
	20	Details of operators performing segmentation	X	☐	☐	6
Pre-processing	21	Image pre-processing details	☐	X	☐	-
	22	Resampling method and its parameters	☐	X	☐	-
	23	Discretization method and its parameters	X	☐	☐	S.Text 3
	24	Image types (e.g., original, filtered, transformed)	X	☐	☐	S.Text 3
Feature extraction	25	Feature extraction method	X	☐	☐	7, S.Text 3
	26	Feature classes	X	☐	☐	7
	27	Number of features	X	☐	☐	7
	28	Default configuration statement for remaining parameters	X	☐	☐	S.Text 3
Data preparation	29	Handling of missing data	X	☐	☐	7
	30	Details of class imbalance	☐	☐	X	-
	31	Details of segmentation reliability analysis	X	☐	☐	6
	32	Feature scaling details (e.g., normalization, standardization)	☐	X	☐	-
	33	Dimension reduction details	X	☐	☐	8
Modeling	34	Algorithm details	X	☐	☐	8

	35	Training and tuning details	☐	X	☐	-
	36	Handling of confounders	☐	X	☐	-
	37	Model selection strategy	X	☐	☐	8
Evaluation	38	Testing technique (e.g., internal, external)	X	☐	☐	8
	39	Performance metrics and rationale for choosing	X	☐	☐	8
	40	Uncertainty evaluation and measures (e.g., confidence intervals)	X	☐	☐	8
	41	Statistical performance comparison (e.g., DeLong's test)	☐	X	☐	-
	42	Comparison with non-radiomic and combined methods	X	☐	☐	8
	43	Interpretability and explainability methods	X	☐	☐	8
**Results**						
	44	Baseline demographic and clinical characteristics	X	☐	☐	10, Table 1
	45	Flowchart for eligibility criteria	X	☐	☐	Fig 2
	46	Feature statistics (e.g., reproducibility, feature selection)	X	☐	☐	Fig. 5, S.Fig. 2
	47	Model performance evaluation	X	☐	☐	10-11, Table 2, Fig. 3, S.Fig. 3
	48	Comparison with non-radiomic and combined approaches	X	☐	☐	10-11, Table 2, Fig. 3, S.Fig. 3
**Discussion**						
	49	Overview of important findings	X	☐	☐	13
	50	Previous works with differences from the current study	X	☐	☐	13-15
	51	Practical implications	X	☐	☐	15-16
	52	Strengths and limitations (e.g., bias and generalizability issues)	X	☐	☐	15-16
**Open Science**						
Data availability	53	Sharing images along with segmentation data [n/e]	☐	X	☐	-
	54	Sharing radiomic feature data	☐	X	☐	-
Code availability	55	Sharing pre-processing scripts or settings	☐	X	☐	-
	56	Sharing source code for modeling	☐	X	☐	-
Model availability	57	Sharing final model files	☐	X	☐	-
	58	Sharing a ready-to-use system [n/e]	☐	X	☐	-
